# Wolf in Sheep’s Clothing: *Clostridioides difficile* Biofilm as a Reservoir for Recurrent Infections

**DOI:** 10.3390/microorganisms9091922

**Published:** 2021-09-10

**Authors:** Jazmin Meza-Torres, Emile Auria, Bruno Dupuy, Yannick D. N. Tremblay

**Affiliations:** 1 Laboratoire Pathogenèse des Bactéries Anaérobies, Institut Pasteur, UMR-CNRS 2001, Université de Paris, 25 rue du Docteur Roux, 75724 Paris, France; jazmin.meza-torres@pasteur.fr (J.M.-T.); emile.auria@pasteur.fr (E.A.); 2 Health Sciences Building, Department of Biochemistry, Microbiology and Immunology, University of Saskatchewan, 107 Wiggins Rd, Saskatoon, SK S7N 5E5, Canada

**Keywords:** *Clostridioides* *difficile* infection, commensal microbiota, dysbiosis, mucosal-biofilm, biofilm inducers, persistence, colonisation resistance, CDI relapsing

## Abstract

The microbiota inhabiting the intestinal tract provide several critical functions to its host. Microorganisms found at the mucosal layer form organized three-dimensional structures which are considered to be biofilms. Their development and functions are influenced by host factors, host-microbe interactions, and microbe-microbe interactions. These structures can dictate the health of their host by strengthening the natural defenses of the gut epithelium or cause disease by exacerbating underlying conditions. Biofilm communities can also block the establishment of pathogens and prevent infectious diseases. Although these biofilms are important for colonization resistance, new data provide evidence that gut biofilms can act as a reservoir for pathogens such as *Clostridioides difficile*. In this review, we will look at the biofilms of the intestinal tract, their contribution to health and disease, and the factors influencing their formation. We will then focus on the factors contributing to biofilm formation in *C. difficile*, how these biofilms are formed, and their properties. In the last section, we will look at how the gut microbiota and the gut biofilm influence *C. difficile* biofilm formation, persistence, and transmission.

## 1. Introduction

The human gastrointestinal tract (GIT) harbors a great diversity of microorganisms known as the gut microbiota [1,2]. The gut microbiota forms complex communities that coexist in an intimate relationship with the host, providing great benefits such as metabolic products and favoring the development of the immune system [3,4]. These gut microbial communities are present as planktonic cells or biofilm communities [5].

## 2. Biofilm Formation in the Gastrointestinal Tract: The Blurry Line between Health and Disease

Biofilm formation is the differential process of planktonic cells to bacterial communities embedded into a thick enclosed-matrix that may or may not be attached to a surface [6]. Biofilm that are not attached to a surface are typically called aggregate biofilms whereas those attached to a surface are called attached biofilms [7]. Biofilm formation in vitro follows a process composed of five stages. The first step includes the initial attachment of cells to the surface [8]. These cells are surrounded by small amounts of exopolymeric material, and many cells are capable of movement. These cells are not yet committed to the differentiation process and can revert to a planktonic lifestyle and exhibit several behaviors such as rolling, creeping, aggregation, and windrow formation [6]. The second stage is the production of extracellular polymeric substances that forms the scaffold for the three-dimensional structure of the biofilm. This allows the cells to stick together and protects bacteria from desiccation, antimicrobials and other stresses [8,9]. The third stage is the early development of biofilm architecture, when many cells alter their physiological processes in order to adapt to a particular niche. The fourth stage is the maturation of the biofilm architecture and the last stage is the dispersion of single cells or aggregates from the biofilm [6].

In the gut, biofilms are embedded in a biopolymer matrix composed of host and microbial material, with the ability to adhere to food particles or mucin aggregates in the lumen, and to a polysaccharide-rich mucus layer lining the gut epithelium [5,10]. Biofilm communities are composed of different species, known as mixed-species or polymicrobial biofilms, that coexist in different microhabitats or metabolic niches and are organized in three-dimensional heterogenous structures [11,12,13].

For a long time, mucosa-associated biofilms have been implicated in human gastro-intestinal diseases [11,14] such as Barrett’s esophagus [15], ulcerative colitis, Crohn’s disease [16,17], *Helicobacter pylori*-induced ulcers [18], and colorectal cancer (CRC) [19,20]. However, recent studies suggest that commensal gut biofilms are present in a healthy colon mucosa; these mucosa-associated biofilms are complex and provide advantageous polymicrobial communities [21,22]. Indeed, interactions between the mucosa-associated commensal microbes and the host favors maturation and activation of the immune system, mucus production, and the growth and development of epithelial cells [12,23]. Furthermore, microbial communities increase colonization resistance against enteropathogens and allow the exchange of nutrients at the epithelial surface [5,12,24]. Disruption or alteration of mucosa-associated biofilms can lead to dysbiosis, allowing adhesion and invasion of epithelial cells by pathogenic bacteria and potentially to inflammation and disease [19,22]. Therefore, a better characterization of the mucosa-associated biofilm communities in the gut, their effect on the host, and their relationship with health and disease is required.

## 3. Gut Biofilm Communities: Location, Organization, and Composition

**Figure 1:**
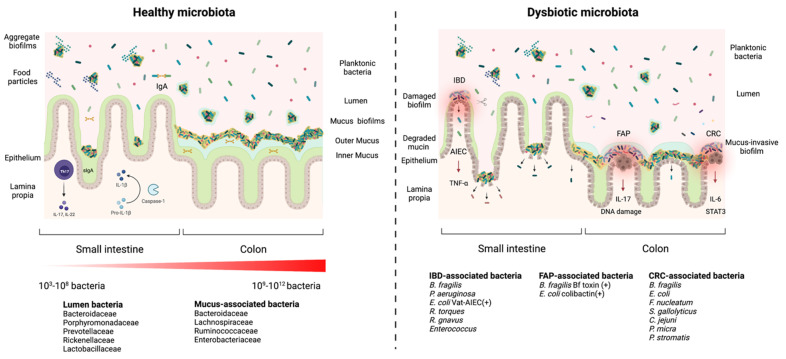
Healthy microbiota biofilms versus a dysbiotic microbiota biofilms. In a healthy microbiota (**left panel**), the microbial density and diversity increase from the stomach to the colon. In the small intestine, biofilms are discontinuous and loose aggregates, while in the large intestine, biofilms are dense, continuous and attached to a uniform mucus layer (attached biofilms). The biofilms in the gut lumen are loosely attached to food particles or encapsulated in mucin (aggregate biofilms). Commensal biofilms facilitate the host barrier function by thickening the mucus layer, regulating the secretion of IgA, stimulating conversion of pro-IL-1β into active IL-1β and inducing the development of Th17 cells. A dysbiotic microbiota (**right panel**) presents (1) damaged mucus-biofilm exposing epithelium cells to luminal content or (2) invasive biofilms where bacteria come directly into contact with the epithelium. Both scenarios expose the intestinal epithelium to pathogens and pathobionts which can trigger an infection. Invasive polymicrobial biofilms could trigger cellular inflammation, abnormal cellular proliferation, increased epithelial permeability (activation of IL-6 and Stat3) in patients with colorectal cancer (CRC), increased IL-17 production and DNA damage in patients with familial adenomatous polyposis (FAP), and inflammatory bowel disease (IBD). Patients’ Adherent-invasive *E. coli* (AIEC) colonize the intestinal mucosa and stimulate the secretion of TNF-α and mucin degradation.

Recent studies are starting to characterize the distribution, composition and characteristics of the gut biofilm communities [23,25,26,27,28,29,30]. The microbial density and diversity increase from the stomach (10^2^–10^3^) to the colon (10^9^–10^12^). In the small intestine, biofilms are found as dispersed, discontinuous and loose aggregates; while in the large intestine, biofilms are dense, continuous and attached to a uniform mucus layer [5] (Figure 1). These variations in biofilm composition and structure along the small intestine and colon are explained by several factors such as chemical and nutrient gradients, as well as compartmentalization of the host immune activity [31]. Furthermore, the composition of biofilm communities in the lumen differs from the communities found in the mucus layer [28,32,33].

Mucus biofilms are associated with mucin or Muc2 glycoprotein [29]. The small intestine harbors a single, tightly attached mucus layer where bacteria are absent; while the colon possess two mucus layers: an inner and an outer layer [34] (Figure 1). The inner mucus layer is directly attached to the epithelium and is very thick and dense, which makes the formation of biofilms difficult [25,35]. The outer mucus layer is less dense and contains a high number of commensal bacteria [36]. The interfold regions contain higher amounts of mucosa-associated microorganisms that use mucins as a nutrient source [37] and as a binding site through specialized structures such as pili [38]. On the other hand, the biofilm populations in the gut lumen are loosely attached to food particles [39] or encapsulated in mucin aggregates in the colon [40] and may present aggregate biofilms.

When the spatial organization of the intestinal microbiota in the mouse ascending colon was studied, it revealed that members of the Bacteroidetes (Bacteroidaceae, Porphyromonadaceae, Prevotellaceae and Rikenellaceae), and Firmicutes (Lactobacillaceae) are found mostly in the lumen whereas members of Firmicutes such as Lachnospiraceae and Ruminococcaceae are found mostly in the interfold regions closer to the gut epithelium [28] (Figure 1). Using laser capture microdissection to isolate mucosa-associated microbes from different regions of the human colon, another study found that the ascending colon was dominated by Proteobacteria, whereas the descending colon was dominated by members of the Proteobacteria and Actinobacteria, followed by Firmicutes [23]. In support of these observations, Fluorescence In Situ Hybridization (FISH) of thin bacterial biofilms found on normal colonoscopy biopsies revealed that the ascending colon is mainly composed of Bacteroidetes, Lachnospiraceae and Enterobacteriaceae, and the descending colon is mainly composed of Bacteroidetes and Lachnospiraceae [19] (Figure 1).

## 4. Health and Disease: Non-Invasive versus Invasive Gut Microbial Biofilms

In a healthy gut, a beneficial microbial biofilm formed by a complex ecological community will interact with the mucus layer and epithelium without invading the epithelia layer. This allows essential functions such as microbiota stability and resilience, which contribute to gut homeostasis and protect against infections [4,5]. Commensal biofilms offer a protective barrier against the proliferation and colonization of enteric pathogens, as well as of opportunistic pathobionts [41]. The resistance mechanisms offered by commensal communities against enteropathogens include the use of bacteriocins and short-chain fatty acids production, which inhibits the growth of pathogens and pathobionts [42,43,44]. Furthermore, commensal bacteria facilitate the host barrier function by thickening the mucus layer, inducing the expression of antimicrobial molecules and regulating the secretion of IgA [45,46,47,48]. Moreover, commensal microorganisms stimulate conversion of pro-IL-1β into active IL-1β [49] and induce the development of Th17 cells in the intestine, allowing protection against pathogens [50] (Figure 1).

On the other hand, when dysbiosis occurs, the physiological conditions in the gut are altered, which affects the organisation of the mucosal biofilm. These changes can result in two possible scenarios: (1) the mucosal biofilm is damaged and forms aggregates of different sizes which leads to the exposure of epithelial cells to luminal content; or (2) an invasive biofilm is formed, bacteria colonize the inner sterile mucus layer and potentially come directly into contact with the epithelium (Figure 1). Both scenarios expose the intestinal epithelium to pathogens and pathobionts which can trigger an infection [5]. For example, changes in diet, such as fiber deficiency, promote the expansion of colonic mucus-degrading bacteria in mice, leading to the erosion of the colonic mucus barrier and facilitating the access to epithelial cells for enteric pathogens that cause colitis in mice such as *Citrobacter rodentium* [51], a surrogate pathogen for enterohemorrhagic *E. coli* (EHEC) and enteropathogenic *E. coli* (EPEC) [52].

Dysbiosis can also lead to invasive polymicrobial biofilms that induce cellular inflammation and abnormal cellular proliferation [19]. Invasive biofilms are associated directly with tumors. A signature of invasive biofilms is the reduction of E-cadherin on the surface of colonic epithelial cells and the high activation of IL-6 and Stat3, which increase epithelial permeability and tissue inflammation [19] (Figure 1). *H. pylori* is able to form biofilms in patients with peptic ulcer disease [18]. *H. pylori* forms biofilm-like microcolonies deep in the stomach glands and interacts directly with gastric progenitor and stem cells in tissues from mice and humans. These gland-associated bacteria accelerate stem cell proliferation and up-regulate the expression of stem cell–related genes, leading to glandular hyperplasia [53].

Bacterial biofilms present in the colon may also alter the host tissue microenvironment and induce metabolic changes in patients with colon cancer, as evident in metabolomic studies demonstrating changes in polyamine metabolite, including an upregulation of *N*^1^, *N*^12^-diacetylspermine. Increased polyamine concentrations are correlated with eukaryotic proliferation, potentially affecting cancer growth, development and progression [54].

Furthermore, invasive polymicrobial biofilms associated with diseases are composed of specific bacterial species or groups. For example, invasive biofilms associated with the colonic mucosa of familial adenomatous polyposis (FAP) patients, an inherited disorder characterized by cancer of the large intestine, were predominately composed of *Escherichia coli* and *Bacteroides fragilis*. These bacteria can secrete oncotoxins named colibactin (ClbB) and *B. fragilis* toxin (BFT), respectively, and these toxins were enriched in FAP patients. Furthermore, mice co-colonized with oncotoxin-producing strains had an increase in IL-17 production in the colon and increased DNA damage in colonic epithelial cells leading to faster onset of tumor [55] creation. Specifically, the BFT toxin triggers a pro-carcinogenic multi-step inflammatory cascade that increases the production of genotoxic oxygen radicals in colonic epithelial cells [56] (Figure 1).

Patients with colorectal cancer (CRC) have a higher number of *Fusobacterium nucleatum* and *Streptococcus gallolyticus* that surround the carcinoma or the adenoma tissues [57,58]. Both bacteria possess virulence factors that stimulate inflammatory and oncogenic responses [59]. Other bacteria that have been found in invasive biofilms in CRC patients are *Campylobacter jejuni, Parvimonas micra*, and *Peptostreptococcus stomatis* [60,61] (Figure 1).

Similarly, invasive biofilms are also associated with inflammatory bowel disease (IBD) such as Crohn’s disease (CD) and ulcerative colitis (UC) [16,17]. In patients with IBD, *B. fragilis* is responsible for more than 60% of the biofilm mass [62]. Another study found a high proportion of pro-inflammatory bacteria on the colonic mucosa of a young patient with ulcerative colitis such as Enterobacteriaceae, *B. fragilis* and *P. aeruginosa* [63]. Adherent-invasive *E. coli* (AIEC) were isolated from ileal biopsies of 36.4% of patients with CD. AIEC colonize the intestinal mucosa, survive and then replicate in epithelial cells and macrophages, which stimulate the secretion of large amounts of TNF-α [64] (Figure 1). Interestingly, AIEC possess a protease called Vat-AIEC that favors mucosa colonization by degrading mucins and decreasing mucus viscosity [65]. Also, an increased prevalence of mucolytic bacterial species such as *Ruminococcus gnavus* and *Ruminococcus torques* were found in CD and UC patients [66]. Furthermore, *Enterococcus* virulence factors were detected in children with IBD, and biofilm production was more frequent among *Enterococcus* strains isolated from children with IBD than in control strains [67] (Figure 1).

Overall, certain intestinal pathologies create an ideal environment which foster enrichment of specific bacterial groups. Bacteria associated with disease will form low diversity biofilm communities that exacerbate underlying conditions whereas bacteria associated with health will form a highly diverse biofilm community that strengthens the natural defenses of the gut epithelium [5]. Development and function of these biofilm communities will be influenced by host factors, host-microbe interactions, and microbe-microbe interactions [5].

## 5. Diversity of Interactions and Phenotypes in the Gut Biofilm Communities

Interactions in mixed-species biofilm communities of the gut can be neutral, positive or negative. Positive interactions are characterized by cooperation, commensalism and cross-feeding, whereas negative interactions are characterized by competition, exploitation and interference [68]. Cooperation involves one species that increases the fitness of another. Cooperation is not always reciprocal; however, if the interaction has a cost for one partner, an indirect benefit should be received for the interaction to be stable [69]. Competition is an indirect interaction between two species competing for a common resource. For example, *Salmonella enterica* induces inflammatory host responses that change the microbiota composition and suppress the microbiota’s growth [70]. In the case of exploitation, one species gains a fitness benefit at the cost to another, and this is also known as predation or parasitism [68]. Interference is a direct interaction where one species affects the fitness of another [68]. Interference includes the use of bacteriocins [71], type V, type VI and type VII secretion systems [72,73,74]. Overall, different types of interactions are occurring in biofilms and these will shape the properties and the special arrangement of biofilm communities.

### 5.1. Competitive and Cooperative Behaviors in a Biofilm

Microbial biofilm communities are spatially organized and can be formed by hundreds of strains and species [75,76]. The arrangement of cells in space is important to define in terms of whether competitive or cooperative behaviors are advantageous in a specific environment [77]. Biofilms can be organized as segregated lineages or mixed lineages. Segregated lineages at a high population density favor cooperative behavior because neighbor cells are almost exclusively clonemates. On the other hand, mixed lineages at a high population density will favor antagonistic interactions, but inter-strains commensalism or mutualism is also observed [75]. Segregation can occur when growing cells are spatially constrained. Mixed lineages can be produced by physical perturbation, diffusive cell movement, spatially homogeneous growth rates, migration and mutualistic cross-feeding interactions [75].

The competitive behaviors will be stimulated when encountering different species, and this strategy favors kin selection [78]. Nutrient scarcity can also lead to bacterial competition against their own kin and other species [79]. Interspecies competition has led to the evolution of competitive strategies [80] such as rapid growth [81], adhesion, matrix production [82], bacteriocins, and toxins production to outcompete neighbors inside a biofilm matrix [83].

The cooperative behaviors can evolve despite their costs [84]. Groups with cooperative behaviors can increase productivity and this increased productivity is sufficient to overcome the costs of cooperation [85]. Cooperative behaviors allow the exploitation of public goods by nonproducing cells or cheating cells [86]. In biofilms, the secretion of extracellular substances allows bacteria to capture nutrients from the environment [21]. For example, cooperative behaviors are present in enteropathogens such as *Vibrio cholerae,* which uses extracellular chitinases to digest its primary food source, the polymer chitin, into N-acetylglucosamine (GlcNAc). In thick biofilms, the chitinases producers confine the goods exclusively to producers. However, fluid flow can wash out the chitin products, allowing cheating cells to benefit from neighbor producer cells [87]. The presence of cheaters alters the structure and properties of biofilms by reducing the population fitness in bacterial biofilms. The population fitness is affected by a decrease in the population growth, a decrease in biofilm thickness, and an increase in the susceptibility of the biofilm to antibiotics [86].

Cooperation could be as extreme as self-sacrifice or altruism where fitness of other members in the biofilm is improved at the expense of the fitness of the producer cells resulting a reduction in the growth rate [69]. Altruism includes the secretion of extracellular enzymes and siderophores that benefit surrounding cells [88]. Also, the prophage-mediated cell death in *P. aeruginosa* allows the differentiation and dispersal of surviving cells inside biofilm microcolonies [89]. Collective behavior is often regulated by quorum sensing (QS), leading to a switch of social behaviors at high densities when specific traits will be more effective and beneficial [90].

Cooperative and competitive behaviors will shape gene expression and, as a result, cellular phenotypes found within a biofilm. This will create sub-populations and heterogeneity within a biofilm.

### 5.2. Phenotypic and Metabolic Heterogeneity Inside a Biofilm

In biofilms, adhesion of cells on a surface results in environmental and cellular heterogeneity [91]. A stratified biofilm has internal gradients with diverse metabolic activities, growth rates, oxygen and tolerance to antibiotics [92]. The bacteria that are in the outer layers of the biofilm have a fitness advantage because oxygen and nutrients are readily available while populations inside the biofilm will have higher tolerance to antibiotics, lower growth rates, low oxygen concentration and low metabolic activity [93]. The creation of nutrient gradients, chemical gradients or waste products within the biofilm can induce the differentiation of cells into diverse phenotypic states and into heterogeneous genotypes [93]. This phenotypic and genetic diversity allows task differentiation better known as the division of labor. This is defined by bacteria specializing in performing different tasks during their cooperative interactions [91]. For example, the biofilm matrix of the soil bacterium *Bacillus subtilis* consists of two major components: exopolysaccharides (EPS) and the cell wall associated protein TasA. Clonal groups of *B. subtilis* segregate phenotypically into three subpopulations composed of matrix non-producers, EPS producers and generalists that produce EPS and TasA. This allows maximum group productivity [94].

As a consequence of environmental heterogeneity, biofilm cells exhibit different ranges of phenotypes and metabolic capabilities than their planktonic counterparts [95,96]. This can be observed among the human gut bacteria found in biofilms, which are more efficient at digesting polysaccharides and the main fermentation product is acetate, whereas nonadherent populations are more efficient at digesting oligosaccharides and the main fermentation product is butyrate. This demonstrates that both communities are metabolically different [97].

The properties of in vitro biofilm may not, however, represent the biofilms observed in vivo because these biofilm communities appear to be smaller and do not share the 3D structure of in vitro biofilms [98]. In vitro models have limits when used to study infectious biofilms because in vivo biofilms seem to be deprived of oxygen and nutrients, are embedded in host derived components, such as pus and wound-bed slough, and are often surrounded by inflammatory cells [99,100]. Indeed, in vivo studies demonstrated that gene expression patterns and metabolic adaptations from human infection samples greatly differ from those obtained under laboratory conditions [101,102]. For in vivo biofilms, decreased virulence, and increased antibiotic resistance and persistence were observed in cystic fibrosis patients with chronic *P. aeruginosa* lung infections [103]. There is a great need to improve techniques and strategies to study in vivo biofilms, but there is also a need to implement new in vitro models that better mimic the in vivo conditions. This would help reduce the discrepancies and improve the biological relevance of in vitro studies.

## 6. Diverse Gut and Microbiota-Derived Signals Induce Biofilm Formation in Commensal Bacteria and Enteropathogens

The transition from a planktonic state to sessile growth is regulated by multiple steps and regulation cascades, and includes QS-dependent genes, the type IV pili (T4P), and the flagellum [104,105,106]. Biofilm formation is also guided by several environmental signals, which include mechanical signals, nutritional and metabolic cues, inorganic molecules, osmolarity, the presence of antimicrobial molecules, quorum-sensing derived signals, and host-derived signals [107].

Bacteria can initiate the transition from a planktonic state to biofilm in vivo to improve their survival against harmful conditions present in the host, to exploit a nutrient rich area that facilitates colonization, or to use the cooperative benefits of multicellular structures [108]. Biofilm formation can be controlled by stress response regulators that are activated by different stresses present in the host such as nutrient limitation, iron deprivation, sub-inhibitory concentrations of antibiotics, and osmotic stress [109,110,111]. Specific environmental conditions such as calcium concentration can increase the second messenger c-di-GMP concentrations that could trigger biofilm formation [112]. In some cases, biofilm formation is dependent on the nutritional conditions that will trigger metabolic adaptation and thus stimulate biofilm formation [106]. In the next section, we will focus on host-derived signals that induce biofilm formation in gut commensal bacteria and enteropathogens.

### 6.1. Host-Derived Factors and Biofilm Formation

Bile salts present in the intestinal tract of the host can induce biofilm formation in several enteropathogens and improve their survival against the toxic effects of bile [113]. Bile salts promote biofilm formation in *V. cholerae* by increasing the intracellular levels of c-di-GMP, which are caused by an increase in c-di-GMP synthesis by 3 diguanylate cyclases (DGCs) and decreased expression of one phosphodiesterase (PDE) [114]. The enteropathogen *Shigella flexnerii* also forms biofilm in response to the presence of deoxycholate (DOC), and this is mediated by the secreted protein IcsA, which is involved in cell-cell contacts and aggregative growth [115]. Similarly, vancomycin-resistant *Enterococcus* (VRE) is able to form biofilms in the presence of physiological concentrations of bile acids, which facilitates colonization and persistence. In VRE, the ability to form biofilms in response to bile salts is controlled by the histine kinase YycG/Walk of the WalRK two component system and the response regulator LiaR of the three-component regulatory system LiaFSR [116]. Likewise, *B. fragilis* treatment with bile salts increased bacterial co-aggregation, adhesion to intestinal epithelial cells and biofilm formation [117]. Exposure to bile salts induced morphological and transcriptional changes in *B. fragilis*, including overproduction of fimbria-like appendages and outer membrane vesicles, and increased expression of genes encoding RND-type efflux pumps and the major outer membrane protein, OmpA [117].

Additionally, *Acinetobater baumannii*, *Cronobacter malonaticus,* and *Bifidobacterium* formed more biofilms when exposed to bile salts [118,119,120]. In *Bifidobacterium breve*, bile-salt-induced biofilm formation involved QS, EPS production and eDNA release, and increased its viability when exposed to porcine bile salts [118]. In *A. baumannii*, exposure to bile salts increased expression of virulence factors associated with surface motility, biofilm, and type VI secretion systems, and these are also associated with activation of the QS system [119]. In the case of *C. malonaticus,* bile salts exposure induced an upregulation of the AcrAB-TolC system, but the molecular mechanisms involved in biofilm formation remain unknown [120].

When the commensal microbiota species *B. breve* and *B. animalis* were grown in taurocholic acid or porcine bile, the bacteria bound more effectively to mucin and formed more biofilm but the molecular mechanism is unknown [121]. Similarly, bile salts can induce biofilm formation in the commensal bacteria *Bacteroides thetaiotaomicron,* and this biofilm formation is dependent on the BT3563 DNAse that degrades extracellular DNA in the biofilm matrix [122].

Human secretory IgA (SlgA) appears to facilitate biofilm formation of the normal gut microbiota in vitro and of *E. coli* on the surface of cultured epithelial cells [123]. SlgA is a key factor that allows agglutination of bacteria and prevents their translocation to the gut epithelial cells, a process known as immune exclusion [124]. It was observed that mucin facilitated biofilm formation by *E. coli* by an unknown mechanism [123]. Similarly, type-2 mucin increased bacterial adhesion and biofilm formation in *Listeria monocytogenes*. This is mediated by the cell-surface protein InlL, which binds directly to Muc-2 [125]. Mucins are also used by *C. jejuni* as a signal to modulate the expression of virulence genes such as mucin degrading-enzymes, flagellin A and toxins [126]. Moreover, *C jejuni* is able to use fucose as a carbon source and shows chemotaxis towards fucose. *C. jejuni* biofilm formation decreased in the presence of fucose, suggesting that *C. jejuni* in a biofilm is able to coordinate fucose use based on its availability [127]. Mucus production in the colon is stimulated by the presence of hydrogen sulfide (H_2_S), which also promotes the establishment of biofilms in the GIT. H_2_S not only promoted biofilm formation by human microbiota ex vivo but also reduced the growth of planktonic bacteria [128].

Many studies have reported that several hormones and vitamins can affect biofilm formation and subsequent colonization. These factors include peptide hormones, steroid hormones such as catecholamine, and vitamin K [129]. For example, the hormone epinephrine was found to induce QS in EHEC [130]. In this study, a *luxS* deletion strain, which is unable to produce the EHEC autoinducer AI-3, responded to the host signal epinephrine and activated the expression of genes involved in biofilm formation [130]. Furthermore, *E. coli* biofilm formation is induced by insulin and is increased when glucose is present [131]. Indeed, the presence of insulin increased *E. coli* hydrophobicity and adherence to epithelial cells [132]. The gut commensal and opportunistic pathogen *Enterococcus faecium* can sense and respond to norepinephrine, a human hormone abundant in the gut, by inducing physiological changes, survival and colonization of the host tissues, and biofilm formation [133]. Catecholamines can also increase adhesion and biofilm formation in the enteropathogens *Salmonella enteritidis* and *E. faecalis* [134,135]. The specific molecular mechanisms of bacterial recognition of the hormones and the activation of regulatory pathways leading to increased biofilm formation have yet to be elucidated. Altogether, these studies show that there is cross-signaling between the host and the microbiota to allow maintenance of the gut homeostasis.

### 6.2. Antibiotics Affecting Biofilm Formation

Exposure to sub-inhibitory concentrations of antibiotics can induce or inhibit biofilm formation in bacteria. In *E. faecalis*, sub-inhibitory concentrations of tigecycline decrease biofilm formation [136], but sub-inhibitory concentrations of gentamicin significantly increased biofilm formation [137]. Similarly, sub-inhibitory concentrations of antibiotics that target the cell wall induced biofilm formation in *E. faecalis* [138]. The increase in biofilm formation was associated with an increase in cell lysis, extracellular DNA (eDNA) levels and cell density within the biofilm. This study included a mathematical model that predicted the changes in antibiotic-induced biofilms due to external alterations, showing that perturbations that reduce eDNA or decrease the number of living cells decreased biofilm induction, while compounds that increased cell lysis and cell wall inhibitors increased biofilm formation [138]. Similar results are also observed in gram-negative bacteria. For example, sub-inhibitory concentrations of aminoglycosides induced biofilm formation in *E. coli* [139]. However, sub-inhibitory concentrations of ceftazidime inhibited *E. coli* biofilm formation by increasing the extracellular concentration of indole [140].

Antibiotic resistance and tolerance can be mediated by efflux pumps and recent studies have suggested that efflux pumps may play a role in biofilm formation [141]. In *E. coli*, efflux pump genes such as *isrA* were highly expressed in biofilm bacteria compared to planktonic bacteria [142]. IsrA mediates the transport of the AI-2 signaling molecule involved in QS, suggesting that efflux pumps may play a role in the transport of the AI in *E. coli* biofilms, facilitating QS and promoting biofilm maturation [143]. Other multidrug efflux pumps such as AcrB and MdtABC were also involved in biofilm formation since corresponding mutant strains decreased biofilm formation and antibiotics resistance [144,145]. Similarly, the efflux pumps of *S. enterica* play an important role in biofilm formation. Indeed, the inactivation of efflux pumps inhibited the expression of the *S. enterica* curli, a surface protein filament that is an essential component of the biofilm matrix [146]. It was suggested that efflux pumps are involved in the activation of the regulator of curli gene expression [141]. In *E. coli*, some drug-induced stresses repressed production of curli and thus repressed biofilm formation [147].

### 6.3. Microbiota Metabolites and Biofilm Formation

Microbiota-produced metabolites can prevent infections and influence the host intestinal homeostasis. Among these, short-chain fatty acids (SCFA) are metabolic products of gut commensals from the *Clostridia* genera such as *Ruminococcus* and *Faecalibacterium* [148]. SCFA protect against enteric pathogens [149]. Moreover, SCFA such as butyric acid, acetic acid, lactic acid, propionic acid, formic acid, and valeric acid are implicated in biofilm formation [148]. Butyric acid and propionic acid induced FimA-dependent biofilm formation by the oral bacteria *Actinomyces oris* [150]. In *A. oris,* biofilm formation is mediated by type-2 Fimbriae composed of the shaft fimbrilin FimA and the tip fimbrilin FimB, which mediate co-aggregation and, subsequently, biofilm formation [150]. Likewise, butyric acid increased biofilm formation in *Actinomyces naeslundii* [151]. On the other hand, SCFA can inhibit biofilm formation in several *Salmonella* species [152].

The signal molecule indole is produced in large quantities by gram-positive and gram-negative bacteria and can act as intra-species, inter-species and interkingdom signal. Indole controls several processes including bacterial physiology, virulence, cell cycle regulation, acid resistance, and biofilm formation [153,154]. Indole was able to activate genes involved in polysaccharide production which are essential for *V. cholerae* biofilm formation [155]. Indole was also able to influence the expression of other genes including those involved in motility. In *E. coli,* indole produced by the tryptophanase TnaA from L-tryptophan, and transported mainly by TnaB, acts as an extracellular signal that regulates the expression of adhesion and biofilm-promoting factors [156]. The tryptophanase TnaA is also present in other species such as *F. nucleatum*, where a *tnaB* homolog was also identified and encodes for a low-affinity tryptophan permease. In *F. nucleatum,* the exogenous concentrations of tryptophan increased indole concentration in the supernatant and biofilm formation in a dose-dependent manner [157]. However, other studies have reported inhibition of biofilm formation by indole. For example, indole significantly diminished *L. monocytogenes* biofilm formation and its virulence genes involved in motility, cell aggregation, and EPS production. In addition, indole regulates many genes involved in virulence and global regulatory genes suggesting that *L. monocytogenes* uses indole from the gut microbiota as a signal to adapt to a new environment [158].

### 6.4. Bacterial and Phages Interactions Affect Biofilm Formation

The gut microbiome contains bacteria, fungi, and viruses, including bacteriophages. These can have an important role in shaping the bacterial population in the gut [159]. For example, exposure of *E. coli* strain MG1655 to environmental bacteriophages results in the selection of phage-tolerant sub-populations that show increased biofilm formation. Interestingly, phage tolerant strains produce large amounts of the DNA-binding protein Dps in the outer membrane and fimbria-like structures [160]. In *S.* Typhimurium, phage predation leads to increase in biofilm formation as a result of non-evolutionary mechanisms, but no phage resistance was observed [161]. On the other hand, some phages mediate biofilm dispersal of *E. coli* under high concentrations of the autoinducer AI-2. In *E. faecalis,* the absence of one prophage resulted in reduced dispersion and the absence of several prophages increased biofilm formation and biofilm dispersal upon induction with AI-2 [162]. In *E. coli*, the transcriptional regulator HhA decreases biofilm formation by repressing the transcription of some tRNAs which inhibit fimbriae production by repressing the genes *fimA* and *ihfA.* The repression of tRNAs by HhA leads to cell lysis and biofilm dispersion due to the activation of prophage lytic genes [163].

As mentioned above, the *V. cholera* QS regulates biofilm formation. In *V. cholera*, the factor VqmA_VC_, a cytoplasmic receptor transcription factor, binds the autoinducer 3,5-dimethylpyrazin-2-ol (DPO) and the DPO-VqmA complex activates the sRNA VqmR, which represses genes required for biofilm formation [164]. Interestingly, a *vqmA* homologue exists in the vibriophage VP882 (VqmA_Phage_). When VqmA_Phage_ binds to DPO produced by the host, the active VqmA_Phage_-DPO allows the transcription of the phage protein Qtip that inactivates the cl repressor, a lytic gene repressor, leading to the induction of the phage lysis program. This lysis program is only favoured under high cell density and high DPO concentrations. Thus, high DPO inhibits biofilm formation and favors bacterial dispersal. On the other hand, when the cell density is low, the phage favors lysogeny. Overall, this allows phage VP882 to integrate host-derived information into its lysis-lysogeny decision based on the state of the QS system and cell-density of its bacterial host [165].

## 7. The Case for *C. difficile*

*Clostridioides difficile,* formerly *Clostridium difficile*, is a gram-positive strict anaerobic bacterium which can be found in the gut of various mammals. This bacterium is an opportunistic pathogen that causes infection, with symptoms ranging from mild diarrhea to pseudomembranous colitis. *C. difficile* infections (CDI) are estimated to cause more than 450,000 infections per year in the United States of America (USA), with an annual cost to the health system of approximately $4.8 billion [166,167]. The more severe cases of CDI can be life-threatening, as the 30-day mortality rate is as high as 21% of diagnosed patients, which results in 15,000 to 30,000 deaths annually in the USA [168]. This opportunistic pathogen is currently one of the rising public health threats as more clinical strains become resistant to the usual antibiotic treatments, including metronidazole and vancomycin [169]. Moreover, several risk factors are associated with CDI including antibiotic therapy and hospitalizations as well as age (≥ 65 years) [170]. Thus, *C. difficile* is the most common cause of nosocomial diarrhea [171], and healthcare associated CDI cases represent more than half of all cases [166].

This bacterium is transmitted by spores through the oral-fecal route. Spores are shed by infected patients and are resistant to both disinfectants and oxygen [172]. Ingested spores usually do not germinate and do not cause CDI because a healthy gut microbiota confers colonization resistance, primarily through the action of bile acids [170]. Primary bile acids are secreted by the host and the highest concentration is found in the small intestine, as 95% of the bile acids are reabsorbed before entering the colon. Primary bile salts are known to promote *C. difficile* spore germination into vegetative cells, while secondary bile acids synthesized from primary bile acids by the microbiota generally inhibit germination and *C. difficile* growth [170]. Disruption of the gut microbiota resulting in dysbiosis is thus necessary to decrease secondary bile acid concentrations and increase the primary bile acid levels. Broad range antibiotics like clindamycin are a primary cause of dysbiosis, as most members of the gut microbiota are sensitive to this treatment and it is more likely to affect the bacteria that convert primary bile salts into secondary bile salts. The changes in bile acid concentrations and profiles allow both spore germination in the ileum and survival of vegetative cells in the colon. Once *C. difficile* starts to colonize the dysbiotic colon, it can overgrow and may start to express virulence factors such as enzymes, which can disrupt the gut barrier (collagenase, hyaluronidase), and toxins (TcdA, TcdB, CDT) to access host-derived nutrients [173]. The toxins are secreted into the extracellular medium and are internalized by the epithelial cells through endocytosis. The toxins can escape the acidified endosome either by creating pores in the endosome membrane (CDT) or by inserting and translocating to the outer layer of the endosome [174]. These events deliver active toxins and lead to the disruption of the tight junctions and inhibition of actin polymerization. These actions break the gut barrier apart and cause pseudomembranous colitis and toxic megacolon. Spore formation is also triggered, allowing the spread of the bacterium to new hosts through shedding in the feces.

One of the particularities of CDI is its high rate of recurrence that can reach 64% for healthcare associated CDI and 28% for community-associated CDI [175]. This recurrence can be either from a newly acquired strain or from the initial strain [176]. Relapses were suggested to be linked to spore formation, as a *spo0A* mutant displayed no recurrent infections in a mouse model [177]. Spores were recently shown to enter intestinal cells, allowing them to survive CDI treatments and ultimately leading to a relapse in CDI [178]. However, spores may not be the only cause of recurrence in CDI. For other bacterial infections such as those involving *Staphylococcus aureus* or *P. aeruginosa* [179,180], recurrent or chronic infections are thought to be mediated by biofilm formation. Thus, biofilm formation by *C. difficile* is hypothesized to be a contributor to recurrent CDI [181]. However, *C. difficile* biofilm formation has only received attention since 2012, and the role of biofilm in the infectious cycle has yet to be identified [182,183].

## 8. Biofilm Formation in *C. difficile*

**Figure 2:**
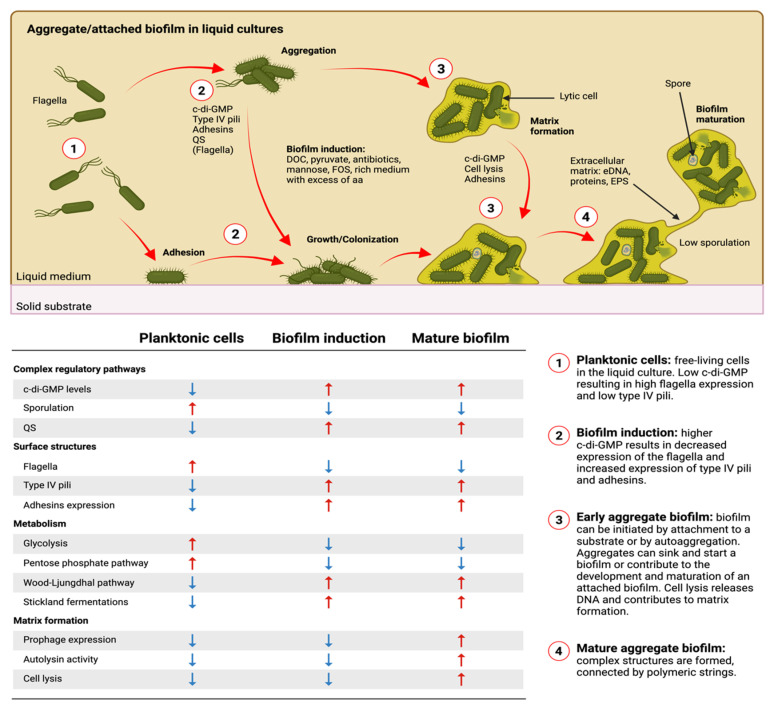
Model for *C. difficile* aggregate/attached biofilm formation in vitro. The first step toward biofilm formation is either attachment of the cells to a surface or cellular aggregation. In both cases a shift in surface structures controlled by rising c-di-GMP levels results in the replacement of the flagella by T4P and adhesins (collagen and fibronectin binding proteins). Autolysin-mediated cell lysis is likely the main mechanism contributing to the formation of the extracellular matrix by releasing chromosomal DNA and cellular proteins in the medium, and exopolysaccharides may be synthesized and contribute to the matrix. Quorum sensing may induce prophage lysis that would also contribute to the biofilm matrix. Furthermore, c-di-GMP levels remain relatively high, ensuring consistent T4P and adhesins expression. *C. difficile* biofilm formation is characterized by a metabolic shift from glycolysis and the pentose phosphate pathway to the Stickland fermentation pathways and the Wood-Ljungdhal pathway, which are less efficient at producing energy. The table summarizes information about the main mechanisms involved in biofilm formation. Up-regulated mechanisms are indicated by the red upward arrows and the down-regulated mechanisms are indicated by the blue downward arrows. Abbreviations: aa: amino acids; QS: quorum sensing; eDNA: extracellular DNA; EPS: exopolysaccharides; DOC: deoxycholate; FOS: fructooligosaccharides.

Interest in *C. difficile* biofilm formation is recent, but it has gathered momentum. From the onset, researchers adapted methods and techniques from other bacterial fields to lay the current foundation. This yielded new tools and models to study *C. difficile* biofilm formation; however, optimization remains an ongoing process. Biofilm formation models and findings for *C. difficile* have been summarized in Figure 2. In the next sections, we discuss the progress in the field.

### 8.1. In Vitro Models, Quantification, and Visualization

Various in vitro models are used to study biofilm formation in *C. difficile*. Closed systems using liquid cultures (cell culture flasks or well-plates) are the most commonly used system because they allow high-throughput experiments [183,184]. However, open system microfermentors characterized by continuous flow are also used, as these are thought to be more physiologically relevant than closed systems in studying gut anaerobic bacteria [185,186]. A chemostat gut model composed of several compartments that represent different parts of the gut with their specific physical and chemical characteristics was also developed and adapted to study gut infections [187,188]. These latter two methods have the advantage of incorporating physiologically relevant conditions of a CDI; however, these types of models can be difficult to set up and require specialized material when compared to their closed systems counterparts. Colony model biofilms are also used to study and image *C. difficile* biofilms [189,190,191]. As a recent study demonstrated, biofilms grown on agar plates had different characteristics in terms of cell-surface protein expression, metabolism, and regulations than biofilms obtained from liquid culture [189].

The typical liquid medium used for in vitro biofilm formation is a rich and complex medium such as Tryptone Yeast extract (TY) or Brain Heart Infusion (BHI) supplemented with yeast extract or casein hydrolysates, L-cysteine and/or glucose [15,18,19,23,29,30]. Biofilms are grown for various periods of time, ranging from 12 h to 7 days [30,31]. Earlier time points allow the study of the early steps of biofilm formation to identify factors involved in the induction of biofilm formation. Later time points allow the study of late stages of biofilm formation to identify factors affecting maturation and dispersion. The use of different time points and growth conditions is one way to demonstrate the plasticity of biofilm formation in *C. difficile* and can provide answers to find pathways involved in biofilm formation. However, the diversity of conditions also makes it difficult to compare studies and draw accurate conclusions. The recent development of a semi-defined medium that supports biofilm formation may help bridge those gaps in knowledge and resolve current discrepancies [192].

When grown in a complex medium, biofilm formation by *C. difficile* is strain dependent and varies greatly [32]. In most studies, the reference strain 630 or its derivative 630Δ*erm* and the clinically-relevant strain R20291 are used as the models for biofilm research, since these are genetically tractable and were the first *C. difficile* strains used for biofilm characterization [15]. In vitro, strains 630Δ*erm* and R20291 form a relatively weak biofilm [193] when compared to most clinical strains, which are strong biofilm producers [31,32,33]. The benefits of having strains forming different levels of biofilms are that conditions inducing or preventing biofilm formation can be easily identified using crystal violet staining. These conditions can be then finely tuned to identify factors that are critical for biofilm formation. Biofilms can be further characterized using different techniques such as microscopy that involves direct staining of the extracellular matrix or cells within the matrix. These images are typically acquired using fluorescence microscopy and specialized stains such as DAPI or BOBO-3 for DNA, calcofluor white for β-1,3 or β-1,4 exopolysaccharides, and Sypro Ruby for proteins [184]. Images acquired using confocal scanning laser microscopes (CSLM) can be analyzed to provide quantitative data on the biofilm. Qualitative images can also be obtained for biofilm formed in well plates and stained with crystal violet [183] or using scanning electron microscopy [182].

### 8.2. In Vivo Models and Clinical Data

The typical infection models for CDI are conventional hamsters or mice whose microbiota was depleted with antibiotic treatments, or germ-free mice (GFM). Imaging of in vivo biofilms can be performed after collecting the samples from the animal models or after a biopsy [172,194,195,196,197]. The intestinal tract samples are fixed, sectioned and stained to visualize the location of the bacteria and epithelial cells. Using this strategy and antibodies against *C. difficile* PS-II and Muc-2, researchers inferred that biofilm-like structures were formed at the mucus layer coating the gut epithelium of GFM, and these biofilms were embedded in mucus and PS-II [196]. In addition, recent studies used clinical isolates and clinical data to identify potential associations between recurrent infections, antibiotic resistance, and in vitro biofilm formation. When tested, the majority of the clinical isolates were strong in vitro biofilm producers [198,199]. Moreover, increased resistance to metronidazole and vancomycin was detected in clinical isolates [200], and these antibiotics are known to induce in vitro biofilm formation [201,202]. Additionally, treatments with metronidazole and vancomycin are associated with a higher rate of recurrent CDI when compared to other treatments, despite metronidazole and vancomycin having higher rates of clinical success [203]. Taken together, these data suggest that biofilms may have an important function in antibiotic resistance in the gut leading to treatment failure, as well as recurrent CDI.

### 8.3. Composition of the In Vitro Biofilm Matrix

Although the exact composition of the matrix of in vivo biofilms might be difficult to determine, precise analysis can be done for the in vitro biofilms. The biofilm matrix is typically composed of DNA, polysaccharides, and proteins, but the composition varies according to species. For *C. difficile*, eDNA is an essential structural biofilm component under all conditions tested [184,192,201,204]. Specifically, adding DNAse I before biofilms were formed prevented their formation [184,201], and DNAse I dispersed pre-formed biofilms [184,204]. Fluorescence staining of eDNA demonstrated its presence in the extracellular space [184]. All these data strongly support the hypothesis that eDNA contributes to the development and structural integrity of *C. difficile* biofilms.

Using fluorescent staining and immuno-staining, the presence of EPS was observed in *C. difficile* biofilms. Based on the localization of the stain, it was suggested that the EPS associated with the bacterial cells were different than those in the intercellular space [190]. For example, the teichoic-like acid PSII, a cell-wall associated polysaccharide, was detected in the intercellular structures as well as on the cellular surface. However, it is not clear how PS-II is organized into the biofilm matrix [201]. Cellulose might also contribute to the biofilm matrix because homologs of the cellulose synthase genes were recently identified in *C. difficile*. Furthermore, a secreted polysaccharide composed of acetylated glucose subunits and hypothesized to be cellulose was detected in culture supernatants [205,206]. *C. difficile* biofilms can be stained with calcofluor white, which is often used to detect cellulose [184,190]. However, calcofluor is not specific for cellulose as it also recognizes β-1,3 or β-1,4 exopolysaccharides, which includes PS-II. Moreover, deletion of the *bscA* orthologue (*ccsA*) encoding a glycosyltransferase involved in cellulose synthesis did not significantly alter DOC-induced biofilm formation [192]. Despite evidence that EPS may contribute to the biofilm matrix, treatment with NaIO_4_ hydrolyzing polysaccharides did not disrupt pre-formed biofilms in vitro [184]. This indicates that EPS are probably not an essential structural part of the biofilm matrix. Further investigations are needed to identify the precise polysaccharides and their role in the biofilm matrix of *C. difficile*.

In addition to eDNA and EPS, proteins were detected in the biofilm matrix of *C. difficile*. Like EPS, proteins are probably not essential for the stability of the biofilm because a treatment with proteinase K did not disperse pre-formed biofilms [184]. However, biofilms formed in a semi-defined medium were sensitive to the proteinase K treatment [192]. Furthermore, adding proteinase K before biofilms are formed prevents biofilm formation [184,201]. A recent systematic analysis of the biofilm matrix showed that the proteins present are intracellular proteins, cell surface, and pathogenicity-associated proteins [204]. Intracellular proteins include transcriptional regulators as well as proteins involved in metabolism. It is likely that the presence of intracellular proteins is the result of cellular lysis. Taken together, the current experimental data suggest that surface or extracellular proteins are essential during the early stages of biofilm formation and may be important for structural stability under specific conditions.

Although proteins and EPS are detected and may play a role in the biofilm matrix of *C. difficile*, eDNA remains a major and essential component that is universal across the growth conditions that were tested.

### 8.4. How Is eDNA Released into the Biofilm Matrix?

Although eDNA is necessary for the formation of *C. difficile* biofilm, it does not appear to be actively secreted given the absence of a secretory system homologous to the one identified in *E. faecalis* [207]. As suggested from the proteomic data of the biofilm matrix, cellular lysis probably supplies the eDNA for biofilm formation. Lysis might occur through four different routes, including (1) prophage-induced lysis; (2) lytic toxin (i.e., programmed cell death); (3) during sporulation; or (4) autolysis. Early lysis may involve prophage production by the vegetative cells, triggered by LuxS and AI2 dependent QS [208]. However, data from our laboratory indicate that deleting the prophages in both strains 630Δ*erm* and R20291 does not significantly change the ability of these strains to form biofilm (Garneau et al., unpublished data). Therefore, the contribution, if any, of eDNA from prophage-induced lysis is probably marginal. A recently discovered type I toxin-antitoxin (TA) system, induced under biofilm growth, [209] has not yet been tested for its contribution to biofilm formation, and no lytic toxin that induces programmed cell death has yet been identified in *C. difficile*.

Recent data revealed a correlation between eDNA content of the biofilm matrix and sporulation frequency [204], while the conclusions relied on biofilm data from a *spo0A*-inactivated strain. Therefore, there is a need for biofilm data for strains lacking specific sigma factors of sporulation (i.e., *sigE*, *sigF*, *sigG* or *sigK*) to provide direct evidence for the role of sporulation as an eDNA contributor. As a preliminary answer, we recently reported that the effect of *spo0A* inactivation was independent of sporulation since inactivation of *sigE* or *sigF* did not prevent DOC-induced biofilm formation (18).

Finally, current evidence strongly supports the idea that autolytic enzymes are probably the main mechanism contributing to eDNA. Indeed, transglycosylases involved in autolysis, such as Cwp19, [210] are more expressed in biofilms than in planktonic cells, [184,189] and inactivation of *cwp19* in the strain 630∆*erm* inhibited biofilm formation in the presence of DOC [184]. Cwp19 requires glucose for its activity [211] and is dispensable for biofilm formation when a different sugar is used (Tremblay and Dupuy, unpublished data). However, *C. difficile* has several autolytic enzymes whose role in biofilm formation has not yet been evaluated.

### 8.5. Surface Structures and Their Importance in Biofilm Formation

In addition to Cwp19, proteins and structures at the cell surface are associated with biofilm formation, and some were found in the biofilm matrix. Among them, T4P are important during the early steps of biofilm formation and dispensable during the later stages [212]. The *pilA1* locus [212], and not the *pilA2* locus [212], appears to be involved in biofilm formation given that deletion of the entire machinery associated with the *pilA1* locus significantly reduced DOC-induced biofilm formation [192]. Furthermore, there is probably a redundancy in the pilin found in the different loci, since deleting a major or minor pilin in any *pil* locus had limited effect or no effect on biofilm formation [185,192,212]. We anticipate that T4P are probably important for biofilm formation in vivo since these surface structures are involved in epithelial adhesion and infections [213].

Since deletion of the *pilA1* locus never fully abolished biofilm formation, adhesion to the substrate and intercellular adhesion, which are important in the early stages of biofilm formation, could be mediated by other surface structures or proteins. In fact, other proteins were identified as contributing to biofilm formation and adhesion to epithelial cells. These include the fibronectin-binding protein FbpA [214], Cwp66, GroEL and the collagen binding proteins CbpA [215] and CD630_28310 [34,49]. In addition, several of these are controlled by c-di-GMP, an important regulator of biofilm formation in *C. difficile* (see below).

One of the surface proteins that had the strongest effect on biofilm formation is the cell wall associated cysteine protease Cwp84. This protease is known to process and cleave the SlpA precursor protein in two sub-units to form the mature S-layer, which is involved in cell adhesion [195,201]. The exact reason why Cwp84 affects biofilm formation remains ambiguous because there are conflicting results in the literature. In strain 630∆*erm*, inactivation of Cwp84 increased biofilm formation [195], whereas in the strain R20291, a 3′end deletion of the *cwp84* gene reduced biofilm formation [201]. The reason for this difference has yet to be resolved, and it remains possible that Cwp84 cleaves other proteins [25]. Overall, S-layer processing is critical for cellular hydrophobicity and proper processing of surface proteins. These could have major effects on cell attachment and biofilm formation.

Another surface structure having a significant impact on biofilm formation is the flagella, which plays an important role as an adhesion factor [216]. There is also an inverse relationship between motility and biofilm formation of clinical isolates, as non-motile isolates were not able to form strong biofilms [193]. In some cases, the flagella appear to affect the maturation of the biofilm. A strain lacking FliC, the main protein component of the flagellum, displayed a wild type phenotype for early biofilm formation, but biofilms appeared to disperse as time progressed [201]. The complemented strain displays the opposite phenotypes: no biofilm production in the early stages, and a normal biofilm production in later stages. Other studies showed that lower glycosylation of the flagellar proteins altered cellular motility, and this was associated with higher levels of biofilm formation. Overall, a non-motile flagellum led to more biofilm biomass than a motile flagellum [216,217]. In the presence of DOC, the absence of FliC or the sigma factor SigD, which regulates flagella expression, did not affect biofilm formation or its kinetics [192]; however, DOC impacts motility and flagella synthesis, and could impact its role during biofilm formation [218].

All of these results are consistent with transcriptional data performed during biofilm formation which show that the genes encoding the flagella components are less expressed, while those of the T4P are more expressed [184,185,192]. Moreover, a recent systematic proteomics study showed that flagella and pili proteins were more abundant in biofilm formed in liquid cultures than in biofilm formed on agar [189]. This confirms that pili and flagella are expressed and produced under biofilm forming conditions but are dependent on the model and conditions used. On the one hand, the absence of a flagella might help *C. difficile* settle to the bottom in static models and a different adhesin would help with early adhesion. The flagella might also act as one of the initial adhesin that triggers a signaling cascade to initiate biofilm formation.

## 9. Regulation of Biofilm Formation

Several regulation pathways and cascades are involved in *C. difficile* biofilm formation, and those involved vary according to the growth conditions. Every study using biofilms from liquid cultures has identified the transcriptional regulators CcpA, CodY, Spo0A and SigL/RpoN as key transcriptional factors involved in biofilm formation [184,185,189,192,219]. CcpA, CodY and SigL/RpoN are global regulators of the metabolism and support the idea that biofilm formation is dependent on a metabolic shift in planktonic cells. Furthermore, the transition phase sigma factor SigH and the master regulator of the sporulation Spo0A are also important for biofilm formation independent of the sporulation process [184]. Additionally, SigH and Spo0A are associated with the metabolism of *C. difficile* [183,192,201]. Other regulators have also been studied in more details and are discussed below.

### 9.1. Are SinR and SinR’ Involved?

*C. difficile* carries two SinR homologs, SinR and SinR’, which interact in a similar manner as SinR and SlrR of *Bacillus subtilis* [219]. In *B. subtilis*, SinR is considered a master regulator of biofilm formation and represses pellicle biofilm formation [220]. When biofilms are induced, Spo0A is phosphorylated, and induces the expression of the SinR antagonist SinI, lifting the repression on gene encoding proteins involved in the synthesis of the biofilm matrix (*eps* and *tasA*) [221]. SlrR and SinR are paralogues that interact to regulate autolysis in *B. subtilis*. In contrast, SinR regulates sporulation and biofilm formation without interacting with SlrR. In *C. difficile*, SinR’ seems to antagonize SinR, as observed for SinI-SinR in *B. subtilis,* and repress the expression of CodY, CcpA and the diguanylate cyclase DccA, leading to a reduction of c-di-GMP levels [222]. Although SinR appears to repress *C. difficile* biofilm formation [219], the effects are not as drastic as those observed in *B. subtilis* [220]. The detail of this repression has yet to be determined.

### 9.2. Is Quorum Sensing Important for Biofilm Formation?

*C. difficile* encodes a homologue LuxS QS system that affects biofilm formation under certain conditions [201,208], while LuxS is dispensable in DOC-induced biofilm formation [192]. LuxS is also associated with prophage induction, and prophage-mediated lysis was suggested as a critical mechanism for biofilm formation. However, under some conditions, prophage induction was high despite the low abundance of LuxS [189]. Based on the presence of *luxS*, it is often assumed that this system acts as an AI-2 based QS. However, there is a lack of genetic evidence for an AI-2 receptor in *C. difficile* and the observed effect might be due to a change in sulfur metabolism [223].

*C. difficile* also encodes an Agr-type QS system that regulates virulence and colonization genes [224]. Strain R20291 has a complete (Agr2; *agrACDB*) and an incomplete (Agr1: *agrD1B1*) Agr system whereas strain 630 only has an incomplete system [224]. In strain 630, the *agr*1 system was not required for DOC-induced biofilm formation [192], and further studies are required to fully assess the role of the *agr* systems.

### 9.3. The Important Role of c-di-GMP in C. difficile Biofilm Formation

C-di-GMP levels are important for *C. difficile* biofilm formation. Indeed, overproduction of c-di-GMP induces autoaggregation and biofilm formation, suggesting that c-di-GMP is critical for the transition from free-living motile state to biofilm communities [225]. Biofilm formation in response to c-di-GMP appears to be controlled through surface proteins regulated by c-di-GMP riboswitches such as those preceding the *pilA1* and *flgB* operons [213,226]. In the case of *pilA1*, the binding of c-di-GMP to type II riboswitch is required for maximum transcription, whereas in the case of the *flgB* operon, the binding of c-di-GMP to the type I riboswitch prevents transcription [213,226]. Similarly, the collagen binding proteins CD630_28310 and CbpA are expressed when c-di-GMP levels are high, which enables the binding of *C. difficile* to collagen. In contrast, when c-di-GMP levels are low, the metalloprotease ZmpI is expressed and exported to the cell wall, allowing cleavage of CbpA and CD630_28310. This cleavage releases the surface proteins from the cell wall and detaches the bacteria from the surface [227,228]. This suggests that c-di-GMP also intervenes for cell detachment or biofilm dispersion.

Despite the rapid effect of the c-di-GMP overproduction, there was no difference in biofilm formation at 72 h [204]. This supports the idea that increased c-di-GMP levels are critical for transition from planktonic cells to biofilm.

### 9.4. Post-Transcriptional Regulation and Phenotypic Heterogeneity in Biofilms

In addition to genetic regulation and riboswitches, post transcriptional regulations by the RNA chaperone Hfq might also influence biofilm formation, since Hfq depletion increases biofilm formation [229]. Factors that control cell homeostasis and cell division or other cellular properties may also influence eDNA release and by extension biofilm production. For example, a strain lacking the Ser/Thr kinase PrkC, known in *C. difficile* to participate in cell wall homeostasis and antibiotic resistance, formed more biofilm in the presence of DOC at earlier time points (24 h) [70]. This strain was also more sensitive to DOC and released more eDNA [230]. Additionally, the absence of the protein chaperone DnaK and the SOS-response regulator LexA in strains 630∆*erm* and R20291, respectively, increase biofilm formation. This is consistent with the fact that flagellum and motility were affected by the absence of DnaK or LexA and might explain the increase in biofilm formation [231,232].

Among the other regulation mechanisms that might also be involved in controlling *C. difficile* biofilm formation, epigenetics may influence biofilm formation, as DNA methylation by the methyltransferase CamA repressed biofilm formation [233]. Phase variation mechanisms controlling flagellar motility, colony morphology, and phosphodiesterases (PDEs) involved in the homeostasis of c-di-GMP and the surface protein CwpV may also influence biofilm formation [234,235,236,237,238]. Indeed, creation of heterogeneity in c-di-GMP levels and surface proteins by phase variation could generate sub-populations within the biofilm, leading to division of labor or dispersion [239].

## 10. What Induces Biofilm Formation?

Despite the identification of genetic regulators, regulation mechanisms and growth conditions controlling biofilm formation, very little is known about specific signals or inducers involved in biofilm induction. Here we present what has been studied so far.

### 10.1. Induction of Biofilm Formation by Antibiotics

In several bacterial species, various stresses induce biofilm formation, in particular antimicrobial and antibiotic stresses [139,240,241,242]. In the case of *C. difficile*, sub-inhibitory concentrations of two antibiotics were found to increase biofilm formation in two clinical strains, vancomycin [201] and metronidazole [202]. Currently, metronidazole and vancomycin are the most commonly used treatments against mild CDI; however, metronidazole is only recommended as an alternative treatment because metronidazole-resistant strains are emerging [200,243]. In addition, berberine chloride, which is used to treat diarrhea, also synergistically induced biofilm formation with vancomycin [244]. Unlike vancomycin and metronidazole, the recently approved antibiotic to treat CDI, fidaxomicin, was able to disrupt in vitro colony biofilms [191]. This is consistent with the reduction in the rate of recurrent CDI with this antibiotic, while metronidazole and vancomycin treatments have limited effects on the rate of recurrent CDI [245]. These data would support the role of biofilm formation as a mechanism for recurrent CDI.

### 10.2. Induction of Biofilm Formation by DOC

Deoxycholate (DOC) is a secondary bile salt synthesized from primary bile salts by the gut commensal bacteria including *Clostridium scindens* [246,247]. DOC has antimicrobial properties, is implicated in colonization resistance against *C. difficile* and was recently shown to induce biofilm formation in strain 630∆*erm* and clinical strains [18]. The induction requires a fermentable carbon source, such as glucose or N-acetyl glucosamine in excess, and cysteine [184,192]. Several genetic determinants were also identified as important for this induction, including an uncharacterized lipoprotein CD630_1687 whose role is currently characterized [184]. Other determinants include the metabolic regulation factors CcpA, CodY, and SigL and the transition phase regulator SigH [184,192]. The need for excess sugars and metabolic regulation factors highlights the importance of metabolism for switching from a planktonic state to *C. difficile* biofilms.

Analysis of biofilm spent culture medium in the presence of DOC allowed for the identification of an excreted metabolite, pyruvate, as important for the induction of biofilms. The role of pyruvate was confirmed when enzymatic depletion of extracellular pyruvate inhibited DOC-induced biofilm formations [192]. Furthermore, medium supplemented with pyruvate can induce biofilm formation without DOC, and glucose is dispensable at higher concentrations of pyruvate [192]. Pyruvate-dependent induction of biofilm formation requires a specific two-component system and at least one transporter (CstA) [192].

It was proposed that DOC induces a metabolic shift leading to overflow metabolism and excretion of excess pyruvate. When glucose is depleted, extracellular pyruvate is detected and imported in the cell, which drives survival during the stationary phase. This provides time for eDNA to accumulate in the medium and enhance cellular adhesion promoting biofilm formation [192].

## 11. The Importance of Metabolism in Biofilm Formation

To study biofilms, two global approaches are generally used: transcriptomics or proteomics analysis. Transcriptomics provides a snapshot of gene expression and non-coding RNA in the recent past and proteomics provides data on the proteins produced during the process. However, neither technique gives information on protein activity. Both omics analyses were performed to study *C. difficile* biofilms. The data collected provided some consensus on surface proteins and regulation factors involved in biofilm formation [185,189,192,195,219]. These analyses were also used to identify metabolic pathways required in biofilms and inferred their properties. However, results are often conflicting because the data were generated from biofilms grown in different conditions.

Another difficulty was that in vitro *C. difficile* biofilms are typically grown in rich and complex media with excess amino acids and fermentable carbohydrates when added to the medium. In closed systems, preferred sources of nutrients will be used first and then depleted. In addition, toxic metabolic waste might also accumulate in the culture medium. Depletion of preferred energy sources will lead to the down-regulation of the metabolic pathways involved and up-regulation of alternative pathways. This has been observed under different conditions that support biofilm formation in *C. difficile* [189,192,219]. For example, glycolysis and the pentose phosphate pathway were downregulated as early as 14 h in cells grown in the presence of DOC despite supplementation of excess glucose [192]. These metabolic pathways remained downregulated after several days of biofilm formation [189,192]. Growth under biofilm inducing conditions also involved butanoate and propanoate fermentation that probably used acetyl-CoA, oxaloacetate and pyruvate, which might be produced by glycine metabolism [192,219]. Furthermore, proteins involved in the Wood-Ljungdhal pathway and glyoxylate shunt were upregulated, as well as the two enzymes, GlyA and TdcB, that convert glycine to pyruvate [219]. In the absence of glucose or DOC, *C. difficile* relies on the activation of the Wood-Ljungdhal pathway to produce energy to induce biofilm formation [23]. Protein degradation and peptide and amino acid intake were upregulated, along with the Stickland fermentation pathways, especially those using branched chain amino acids [192]. Overall, pyruvate production from the available precursors was found to be central for biofilm formation [189,192,219].

Unlike closed systems, continuous flow systems constantly replenish nutrients, and this has a significant effect on the metabolism (Figure 2). Specifically, the Wood-Ljungdahl pathways, fermentation pathways and most Stickland reactions were down-regulated [185]. Instead, glycolysis genes and the pentose phosphate pathway were up-regulated [185]. Interestingly, pyruvate remained a key metabolite but was produced by glycolysis, the pentose phosphate pathway and cysteine metabolism [185]. Unlike the metabolism of biofilms formed in closed systems, pyruvate was mostly used to form acetyl-CoA and then fatty acids to regenerate NAD+. *C. difficile* can also use succinate to restore NAD+ levels. Activation of fatty acid biosynthesis and succinate catabolism are typically required when glycolysis is highly active because it produces more NADH.

Although glucose is often used to support biofilm formation, other carbohydrates can induce biofilm formation as well. Indeed, mannose and fructooligosaccharides, which are toxic at high concentrations, were able to induce biofilm formation in *C. difficile* at sub-inhibitory concentrations [248]. In the gut, *C. difficile* would use mucin-derived sugars such as N-acetylglucosamine and sialic acid. These can be used by *C. difficile* to sustain its metabolism and form biofilms [192]. *C. difficile* colonization is impaired when these sugars are used by other gut microorganisms [249]. Furthermore, mucus-derived sugars act as a chemoattractant for *C. difficile* and direct its movement towards the mucus layer [250]. Taken together, the data reinforce the model of a *C. difficile* biofilm associated with the colonic mucus layer as observed in vivo [196] and in mucus-degrading microorganisms.

## 12. Role of the Microbiota-*C. difficile* Interactions in Biofilm Formation

Enteropathogens are often able to form single and mixed biofilms in the gastrointestinal tract. FISH and 16rRNA sequencing confirmed that *C. difficile* can integrate communities of the cecum, and these communities were associated with the outer mucus layer [194]. The predominant members of these communities are *Bacteroidetes* and *Firmicutes* in which *C. difficile* is in the minority [194].

Bacteria such as *C. scindens* are known to limit *C. difficile* colonization in in vivo models, but it can enhance *C. difficile* biofilm formation in vitro by producing DOC from cholate [184,251]. This indicates a more complex relationship between these two bacteria than previously thought. Depletion of *C. scindens* in the gut during an antibiotherapy promotes CDI by stopping production of DOC. After the CDI is treated, *C. scindens* may produce sub-inhibitory concentration of DOC as its population is restored, and these concentrations could induce biofilm formation by *C. difficile*.

Other bacteria can also enhance biofilm formation when co-cultured with *C. difficile*. Specifically, *Finegoldia magna* and *Fusobacterium nucleatum* enhanced biofilm formation in co-cultures with *C. difficile* [26,182]. The synergy with the latter is based on an interaction between *C. difficile* flagella and *F. nucleatum* adhesin RadD [26]. This interaction is relevant because there is a positive correlation between the presence of *F. bacterium* in the gut and CDI [26]. Additionally, a consortium of *B. thetaiotaomicron*, *B. fragilis*, *S. warneri* and *C. parapsiloris* also supported mixed-species biofilm formation with *C. difficile* [252]. On the other hand, *B. fragilis* co-cultured with other bacteria such as *L. rhamonosus*, *B. longum* and *B. breve* can also reduce *C. difficile* biofilm formation [253].

In addition to bacteria, fungi may play a role in the development of CDI, as recent studies identified a fungus-associated bacteriome affecting this infection. In addition, fungus-associated bacteriome enhanced *E. coli* and *P. aeruginosa* biofilms [254]. Specific metabolic and communication pathways were associated with these microbiomes, and included linoleic acid metabolism and autoinducer-3 mediated quorum sensing, suggesting trans-kingdom communication [254]. Overall, complex interactions from consortia will dictate the outcome of *C. difficile* colonization and biofilm formation and these might be strain-specific and not predictable from dual-species interactions.

## 13. Gut Biofilm: A Shelter against Stresses for *C. difficile*

The ability of bacteria to form biofilms is often associated with stress adaptation and chronic infections. Indeed, the National institute for health (NIH) estimates that 65% of microbial infections and 80% of chronic infections are mediated by biofilms [253]. This is mainly due to the strong competitive advantages offered by biofilms when bacteria are exposed to various environmental challenges [255]. Specifically, cells inside biofilms are less sensitive to antibiotics and host immune responses than planktonic cells. The mechanisms mediating these changes are hypothesized to reflect the expression of biofilm-specific genes and the ability of bacteria to persist in vivo [142,256,257,258].

As observed for other pathogens, *C. difficile* cells grown as biofilms are less sensitive to antibiotics commonly used to treat CDI. For example, *C. difficile* strain R20291 had a 10 times higher survival rate than planktonic cells when exposed to vancomycin [201]. This was confirmed with various *C. difficile* clinical strains using different biofilm-forming conditions [184,187]. Similarly, *C. difficile* clinical isolates grown as biofilms were 100-fold more tolerant to metronidazole than cells grown in liquid culture [184,187]. Unlike fidaxomicin, vancomycin and metronidazole were less effective in penetrating and killing vegetative cells within established biofilms and cannot reduce the number of spores inside a biofilm [191]. This is also consistent with the fact that fidaxomicin is more effective at reducing recurrent CDI rates than vancomycin and metronidazole. Recently, a larger set of antimicrobial compounds, including thuricin CD, tigecycline, teicoplanin, rifampicin, and nitazoxanide were assayed for their activity against biofilms formed by a collection of *C. difficile* strains. Combined antimicrobial therapies were more effective against biofilms of strain R20291 than treatments with a single antibiotic. Furthermore, sensitivity to different antimicrobial drugs or combinations was strain-dependent and varied according to the amount of biofilm formed by each strain [259].

Based on the observation described above and studies with other bacteria, the biofilm matrix probably mediates the decrease in antibiotic sensitivity of *C. difficile* biofilms. The biofilm matrix can act as a physical barrier that reduces penetration of antibiotics resulting in a decrease in the antibiotic concentration inside the biofilm [201,260]. This is supported by recent data where DNase I treatment increased the effectiveness of vancomycin against biofilm formation [204]. Multiple mechanisms are also involved in the decreased antibiotic sensitivity of biofilms, and these will be dependent on the bacterial species and the type of antibiotic used. For example, the low penetration of antibiotic resulting in sub-inhibitory concentrations can induce expression of genes mediating antibiotic resistance [261,262]. Additionally, low metabolic activity of cells in the deeper layer of the biofilm can reduce antibiotic killing activity. Moreover, the presence of antibiotic-degrading enzymes in the biofilm matrix can decrease the antibiotic concentration within biofilm [260,263]. Finally, the establishment of persisters could lead to the creation of microbial reservoirs that are protected from antibiotics inside biofilms. Persisters can survive antibiotics treatment by adapting their metabolism and/or promoting the appearance of antibiotics resistance through the spread of resistance plasmids [264]. It was demonstrated that *C. difficile* formed persister-like cells in response to antibiotic treatment [265]. The mechanisms described above are all potential explanations for the change in sensitivity to biocidal agents for *C. difficile* biofilms, but further studies are required.

In addition to reduced antibiotic susceptibility, *C. difficile* biofilms are less sensitive to oxygen [183], DOC and antimicrobial peptides [184], but these resistance mechanisms are not yet understood. Altogether, current evidence suggests that biofilms might play an important role in the adaptive response and persistence of *C. difficile* in the gut leading to asymptomatic carriage and relapse after antibiotic therapy.

## 14. Persistence in the Gut: Spores, Biofilms, or Both?

Initially, persistence *of C. difficile* in the gut of mice was associated with the formation of spores. It is generally accepted that spores will form during the infection, survive the antibiotic treatment, and germinate once the antibiotic treatment is ceased. Evidence for this model is partly based on studies demonstrating the ability of spores to enter epithelial cells [178] and the inability of a non-sporulating *spo0A*-inactivated strain to persist in the intestinal tract of mice and cause relapses [177]. However, inhibiting spore entry into epithelial cells only delayed relapsing CDI [178], and inactivation of *spo0A* has pleiotropic effects on metabolism and biofilm-formation [183,266]. Overall, these findings suggest that *C. difficile* persistence and recurrence may not be solely dependent on spores.

*C. difficile* persistence in the intestinal tract may be driven by multispecies biofilm communities, which may contribute to recurrence of CDI [181]. In support of this mechanism, several studies have demonstrated that multi-species biofilms formed by the gut microbiota can harbor *C. difficile* and act as a reservoir for recurring infections [182,187,190]. Furthermore, we showed that *C. difficile* can form dual-species biofilm when grown with *C. scindens*, a bacterium that converts primary bile salts to secondary bile salts, and in the presence of cholate [184]. Biofilm-like structures have also been observed on the epithelium of hamsters and mice, or on the cecum mucus layer of mice [172,196,197]. Overall, these studies provide good evidence that *C. difficile* can form biofilm communities in the gut; however, there is a lack of direct evidence that these biofilm communities are a source of vegetative cells and spores for recurring *C difficile* infections.

**Figure 3:**
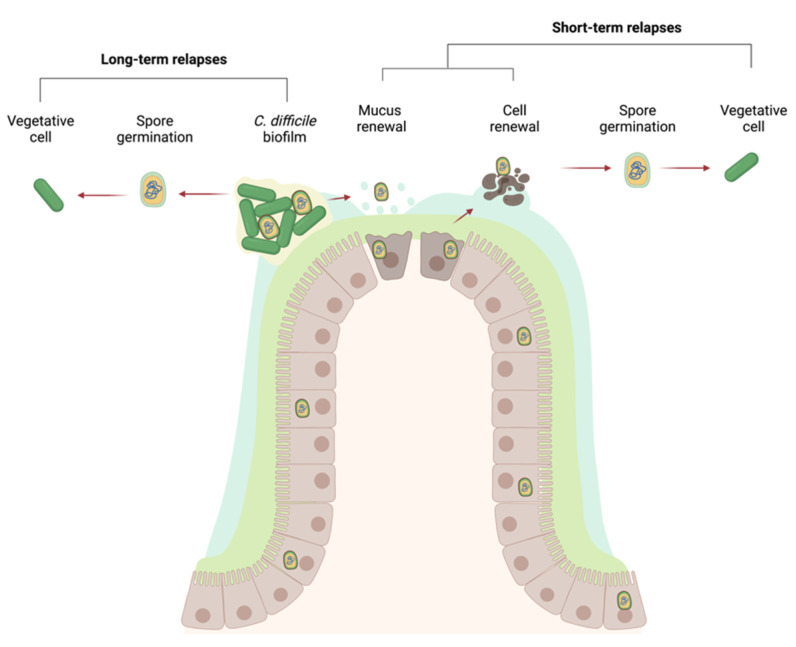
Proposed model for the persistence of *C. difficile*. In this model, spores (circles) and biofilms contribute to short term and long-term relapses, respectively. Spores encased in mucus, biofilm communities or engulfed by epithelial cells, would eventually be eliminated by the renewal of the mucus and epithelial cells. The vegetative cells (rods) would keep a small viable population resulting in a biofilm that would be resistant to renewal of the mucus layers and epithelial cells. Sporulation could occur in the deeper layers of the biofilm, keeping a continuous supply of spores and leading to long-term relapses.

Based on the current evidence, it is possible that both sporulation and biofilm formation are important for persistence and recurrence, but their contribution could be different (Figure 3).

Spores are dormant and passive passengers that would not react or adapt to changing conditions, whereas biofilms are composed of vegetative cells that can actively adapt and maintain a population under changing conditions. Because of their state, spores are at risk of being cleared by the normal physiological processes occurring in the gut (Figure 3). Specifically, epithelial cells are renewed every four to five days under healthy conditions [267], and spores inside epithelial cells could be quickly shed and eliminated within a week. Furthermore, the inner mucus layer of the colon is renewed every 1–2 h [268] and this continuous renewal could eliminate spores that are trapped within the mucus and/or biofilm communities (Figure 3). However, the microbiota composition and diversity, the inflammation processes and antibiotic treatments will affect these natural protective mechanisms and their renewal process [269]. It is more likely that spores participate in short-term recurrence than those that occur weeks later. Vegetative cells in a biofilm community would be the population responsible for long-term relapses (Figure 3). Multiple factors would contribute to population maintenance in the outer mucus layer, including a generation time equivalent to the rate of mucus renewal, the colonization coverage by *C. difficile* and the mucolytic activity of the microbiota. Perturbation to this equilibrium could affect generation time leading to localized population collapse and, if it becomes generalized, could lead to eradication of *C. difficile* from the gut via the normal protective mechanism.

A well-established biofilm community would be able to keep a small viable *C. difficile* population, but toxin production and population blooms would be kept in check by the microbiota. In this scenario, the vegetative cells would continuously generate spores to replace those eliminated by mucus renewal (Figure 3). This renewing stock of spores could contribute to a relapse after an antibiotic treatment.

Overall, the current evidence does not support an exclusive role for sporulation or biofilm formation as the mechanism behind persistence or recurrence for *C. difficile*. To move forward, there is a need for studies that investigate recurrent CDI with strains lacking genes only affecting sporulation, namely *sigE*, *sigF*, *sigG,* and *sigK*. This would help define the role of spores in recurrent CDI given that we have evidence that, unlike *spo0A* or *sigH*, *sigE* and *sigF* do not affect biofilm formation in the presence of deoxycholate [184,192]. It will, however, be more difficult to define the role of biofilms in recurrent CDI. Recent studies provide evidence that biofilm formation is dependent on metabolism and excreted metabolites [192,270]. Therefore, we think there is a need to refine our view on the infectious cycle of *C. difficile*.

## 15. Refining the Infectious Cycle of *C. difficile*: Metabolic Landscape as a Determinant of Biofilm Formation, Pathogenesis or Sporulation

**Figure 4:**
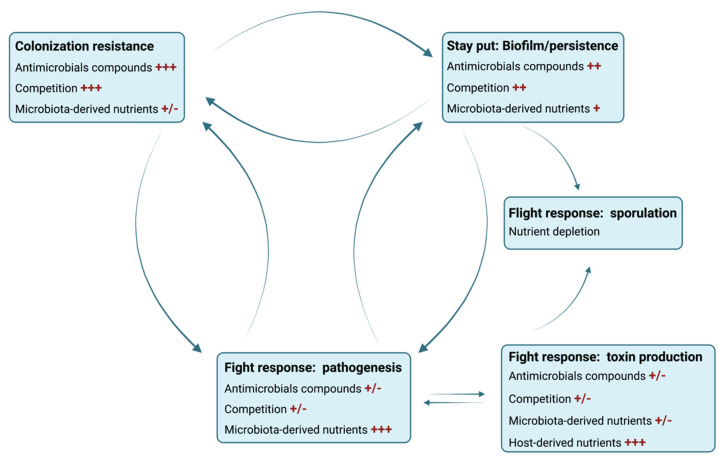
Proposed life cycle of *C. difficile* based on the metabolic landscape of the gut. The metabolic landscape is determined by 3 variables: competition, availability of microbiota-derived nutrients and availability of host-derived nutrients. Concentration of antimicrobial compounds and the metabolic landscape will determine the *C. difficile* growth rate and toxin production. Specifically, biofilm-persistence is a response to ecological competition caused by restriction in nutrient availability due to moderate levels of competition from the resident microbiota. Pathogenesis is a response to nutritional stress caused by a decrease in the availability of nutrient due to overgrowth. Sporulation is a response to starvation due to the depletion of nutrients in the gut or localized in the deeper layer of the biofilm. +++ high, ++ Moderate, + low, +/− very low.

During an infection, metabolic adaptation is an important aspect that will shape the outcome of colonization and symptoms. The composition of the microbiota will greatly influence the concentration of available nutrients that can favor or prevent *C. difficile* colonization [271], and recent in silico modeling suggests that virulence and sporulation have specific metabolic intake and output [272]. Furthermore, computer-generated models of CDI support the idea that changes in specific nutrients such as amino acids and glucose, combined with a decrease in butyrate and an increase in acetate, drive disease progression and recurrence [273,274]. Therefore, the life cycle of *C. difficile* should be centered around the metabolic landscape of the gut rather than microbiota dysbiosis. We suggest that metabolic intake should lead to three different outcomes when colonization is successful: stay put and under the radar (i.e., biofilm/persistence), fight (i.e., virulence/toxin production), or flight (i.e., sporulation) (Figure 4).

For the stay put and under the radar outcomes, the microbiota have to produce sub-inhibitory concentrations of antibacterial compounds that prevent *C. difficile* bloom, but there would be minimum competition for microbiota-derived metabolites essential for *C. difficile* survival, such as mucus-derived sugars, branched-chain amino acids and proline. Sub-inhibitory concentration of antibacterial compounds like DOC would trigger a metabolic adaptation in *C. difficile* to use the available metabolites such as pyruvate to sustain its viability. Therefore, *C. difficile* could persist in the gut by forming a multispecies biofilm associated with the mucus layer. Under these conditions, *C. difficile* would be difficult to detect in feces because few bacteria would be in the lumen and the colonization would be asymptomatic due a lack of toxin production.

The fight response would be induced by a major change in the microbiota such as an antibiotic-treatment. These changes would remove competitors and the production of antibacterial compounds. *C. difficile* would grow unchecked and bloom which would deplete microbiota-derived nutrient sources. This forces *C. difficile* to use its toxin to induce inflammation and change the nutritional landscape to its advantage. This excludes competition and makes certain host-derived nutrients available, such as sorbitol [173,275,276]. These host-derived nutrients would eventually be depleted and this, in addition to elevated oxygen concentration and the immune response, would induce the flight response (i.e., sporulation). Alternatively, sporulation could be induced in specific biofilm subpopulations that are in the deeper layers of biofilm because these bacteria may have restricted access to microbiota-derived nutrients. The spores produced in the biofilm could then contribute to recurrence and transmission.

In summary, there is a need to refine our view on the *C. difficile* lifecycle and it should be centered on the metabolic landscape of the gut. Specifically, virulence and toxin production should be viewed as a response to nutritional stress, sporulation as a response to starvation, and biofilm formation as a response to ecological competition.

## Data Availability

No new data were created or analyzed in this study. For unpublished results, data are available on request from the corresponding authors.
